# Antimicrobial Chemokines

**DOI:** 10.3389/fimmu.2012.00276

**Published:** 2012-09-11

**Authors:** Sunny C. Yung, Philip M. Murphy

**Affiliations:** ^1^Laboratory of Molecular Immunology, National Institute of Allergy and Infectious Diseases, National Institutes of HealthBethesda, MD, USA

**Keywords:** chemoattractant, immunology, G protein-coupled receptor, mucosa, microbiome

## Abstract

Chemokines are best known for their classic leukocyte chemotactic activity, which is critical for directing the immune response to sites of infection and injury. However, recent studies have suggested that at least some chemokines may also interfere with infectious agents directly. Antimicrobial chemokines tend to contain amphipathic alpha helical secondary structure, and broad-spectrum activity against both Gram-positive and Gram negative bacteria, as well as fungi. Conversely, several bacteria have been identified that possess mechanisms for specifically blocking the antimicrobial activities of chemokines. Although the precise mechanisms by which chemokines and microbes disarm one another *in vitro* remain unknown, there is now emerging evidence *in vivo* that such interactions may be biologically significant. More research will be needed to determine whether chemokines with direct antimicrobial activity may be translated into a novel class of antibiotics.

## Introduction

Chemokines comprise a family of phylogenetically related, small proteins whose main shared function is to recruit leukocytes to sites of inflammation and infection (Murphy, 2008). In addition, some chemokines are important for tissue repair, organ development, and cancer. Many chemokines have been shown to have direct antimicrobial properties *in vitro*, however the *in vivo* significance of this has not been established.

**Figure 1:**
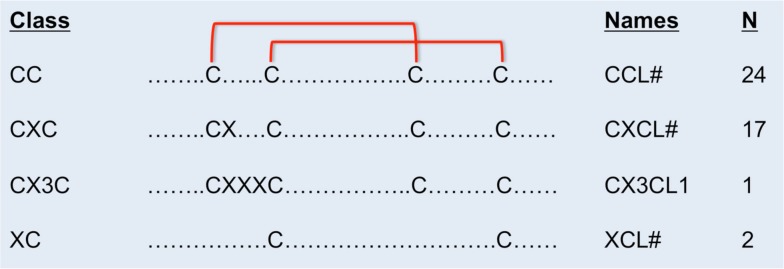
**Cysteine disulfide bonds in different classes of chemokines**. In CC chemokines, the first two conserved cysteines are adjacent. The first and the third, and the second and the forth conserved cysteines form disulfide binds. In CXC chemokines, the first two conserved cysteines are separated by one amino acid. In the CX3C chemokine, three amino acids separate the first two conserved cysteines. There are only two conserved cysteines in the XC class of chemokines. The name and number of human members in each class of chemokines are on the right.

Chemokines are classified according to the number and arrangement of conserved cysteine residues (Murphy et al., 2000). In humans, all but two chemokines contain four conserved cysteine residues with disulfide bonds linking C1–C3 and C2–C4 (Figure 1). The first two cysteines are adjacent (CC motif, *n* = 24), separated by one amino acid (CXC motif, *n* = 17), or separated by three amino acids (CX3C motif, *n* = 1). The XC chemokines (*n* = 2) have only two cysteines. Sequence identity is <30% between members of different classes, but within the same class, sequence identity ranges from ∼30 to 99% within species. Most chemokines are cationic and ∼7–12 kDa in molecular weight.

Although some chemokines are constitutively expressed, the majority are induced under inflammatory conditions, often in response to pleiotropic cytokines. Inducible chemokines are involved in host defense (both innate and adaptive immunity), and in acute and chronic inflammation. There are chemokines specialized for recruitment of neutrophils (CXCL1, CXCL2, CXCL8), monocytes (CCL2, CCL7, CCL13), Th_1_cells (CXCL9, CXCL10, CXCL11), Th_2_ cells (CCL17 and CCL22), Th_17_ cells (CCL20), and other immune cells to sites of trauma, ischemia, and infection (Olson and Ley, 2002; Singh et al., 2008). During the course of an infection, pathogens may be exposed to many chemokines.

## History of Antimicrobial Chemokines

Antimicrobial activity was discovered for chemokines in the year 2000 with the identification of truncated forms of CXCL7 as platelet microbicidal proteins (Krijgsveld et al., 2000). Blood platelets have been known to release antibacterial proteins upon thrombin activation *in vitro* (Yeaman, 1997). These antimicrobial proteins, designated as thrombocidins, had both antibacterial and antifungal properties and were identified as C-terminal deletion products of CXCL7 by protein sequencing. Within the next several years, other investigators discovered additional chemokines (CXCL9, CXCL10, CXCL11, CXCL6, CXCL14, CCL20, and CCL28) that had antimicrobial properties (Cole et al., 2001; Hieshima et al., 2003; Yang et al., 2003; Linge et al., 2008; Maerki et al., 2009). Since these chemokines can be induced during inflammatory conditions, they may act as the first line of defense against pathogens.

## Antimicrobial Assay

**Figure 2:**
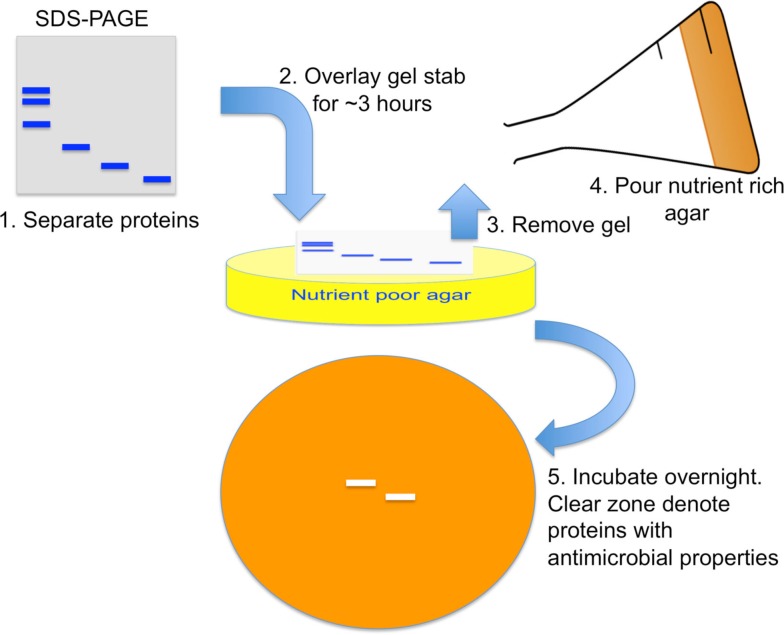
**Antimicrobial gel overlay assay**. Proteins within a lysate with known antimicrobial properties are separated by SDS-PAGE. A nutrient poor agar with low sodium concentration is mixed with pathogen to make up the bottom layer. A piece of the SDS-PAGE gel stab is placed on top for several hours for proteins to diffuse from the gel onto the agar. The gel is then removed and a nutrient rich agar placed on top. After overnight incubation, a clear zone with no pathogen growth represents locations of antimicrobial proteins.

Antimicrobial effects of chemokines were discovered *in vitro* with a gel overlay assay and quantified by microdilution comparison, or radial diffusion methods (Krijgsveld et al., 2000; Cole et al., 2001; Yang et al., 2003). In the gel overlay assay, a lysate with known antimicrobial properties is separated by SDS-PAGE. Next, the pathogen to be tested is resuspended in nutrient poor agar and poured onto a tissue culture plate (Figure 2). Then, a portion of the SDS-PAGE gel is cut and placed on top for several hours to allow transfer of proteins from gel to agar. The gel is removed and a nutrient rich agar is placed on top. After overnight incubation, band areas without pathogen growth represent the location of antimicrobial proteins.

For quantitative analysis, microdilution comparison and radial diffusion methods have been used. For microdilution comparison, a known amount of a pathogen is incubated with increasing concentration of antimicrobial chemokine in a nutrient poor broth. After several hours, the number of viable organisms is determined by serial dilution on nutrient rich agar plates. Percent inhibition is determined by the difference of viable organisms between chemokine treated and untreated samples using the formula: [( w/o chemokine) − ( w/chemokine)/( w/o chemokine)] × 100.

In the radial diffusion assay, organisms are mixed into nutrient poor agar. Then, 3 mm diameter wells are punched out of the agar. Next, a known concentration of chemokine is added to the wells and incubated for several hours to allow protein diffusion. Finally, a media rich agar is overlay on top. After overnight incubation, the diameters of clear zone surrounding the wells are measured and used to calculate the potency of the chemokine against the test organism.

## Salt Dependence of Chemokine Antimicrobial Activities

The nutrient poor agar and broth, described above, all contained low ionic concentrations, with most assays having 10 mM of Na^+^. As the Na^+^ concentration is increased to 100 mM, the antimicrobial activity of chemokines diminishes or even disappears. Table 1 lists the reported electrolyte concentration of different fluid compartments in humans. In particular, sweat and mucosal secretions, such as saliva, have low ionic conditions that would favor antimicrobial activities. Therefore knowing whether antimicrobial chemokines are secreted into these fluids is important. Indeed, several chemokines have been reported in sweat (CXCL8 and CCL2) and in saliva (CXCL8; Jones et al., 1995; Yang et al., 2005). Theoretically, chemokines may inhibit growth of organisms on the skin and mucosal surfaces, provided they are in high enough concentrations for antimicrobial activity.

**Table 1:** **Electrolytes concentration and major components in different human secretions**.

Fluid	Sodium (mmol/L)	Chloride (mmol/L)	Potassium (mmol/L)	Bicarbonate (mmol/L)	Phosphate (mmol/L)	Creatinine (mmol/L)	Others (mmol/L)
Serum^1^	136–145	98–106	3.5–5.0	23–28	0.97–1.45	7–13	
Sweat^2^	15–90	<40	0.002	2–40			
Saliva^3^	2–50	5–40	10–36	25	1.4–39		
Tears^4^	120–177	87–137	13–24				
Nasal^5^	100–140	100–160	10–25				
Urine^6^	10–200	15–200	10–0	<0.1–low		133–221 mmol/kg per 24 h	
Bile^7^	200–260	10–60	5–8	10–60			Taurocholate 100–260
Gastric juice^8^	100	280	15				

Pancreatic juice^8^	150	40	5				

Intestinal fluid^8^	150	100	5				

Stool^8^	44–112		29–147				
Diarrh^8^	3–139		15–115				
Milk^9^	5–8	9–13	11–15				Lactose 193–207
PBS	137	139.7	2.7		10		

*Listed are the major ions found in human secretions. Major compounds such as creatinine, taurocholate, and lactose in urine, bile, and milk are also indicated*.

^1^MAKSAP 14 Normal Laboratory Values. American College of Physicians, Copyright 2005. All rights reserved

*^2^Bulmer and Forwell (1956), Kaiser et al. (1974)*.

*^3^Chicharro et al. (1994)*.

*^4^Lew et al. (2005)*.

*^5^Cavaliere et al. (1988), Cavaliere et al. (1989)*.

*^6^Moritz (2008)*.

*^7^Wheeler et al. (1960)*.

*^8^Shiau (1987)*.

*^9^Koo and Gupta (1982), Wack et al. (1997)*.

## Concentration of Chemokines Needed for Antimicrobial Activity

For most antimicrobial chemokines, micromolar concentrations are needed for pathogen killing (Krijgsveld et al., 2000; Cole et al., 2001; Yang et al., 2003; Maerki et al., 2009; Yung et al., 2011). In contrast, leukocyte chemotaxis *in vitro* typically requires 1000-fold lower concentrations of chemokine (Berkhout et al., 1997; Siveke and Hamann, 1998). A non-comprehensive list of chemokine concentrations found constitutively in different body fluids is listed in Table 2. The most extensive survey has been obtained by bronchiolar alveolar lavage (Pacheco-Rodriguez et al., 2009). In general, naturally occurring chemokine concentrations are in the picomolar to nanomolar range. However, two chemokines produced by platelets are found at micromolar concentrations in serum (Brandt et al., 2000). CXCL4 (also known as platelet factor 4) was the first chemokine ever to be characterized. It binds to the receptor CXCR3B, a splice variant affecting the N-terminus. The other is CXCL7 (also known as neutrophil-activating peptide 2). Both chemokines are released during platelet activation. From the table, it is apparent that many chemokines are made constitutively and secreted into serum/plasma, sweat, tears, saliva, and breast milk.

**Table 2:** **Chemokine concentrations in different body fluids**.

Chemokine		Size MW (kD)	Serum or plasma nM	BAL (nM)	Sweat (nM)	Tears (nM)	Saliva (nM)	Parotid Secr (nM)	Breast milk (nM)
Family	Member								
CXC ELR+	CXCL1	7.8	0.01*D*	3.07*T*					0.4*I*
	CXCL2	7.9	0.03*X*						5.96*I*
	CXCL3	7.9	0.09*P*						0.62*I*
CXC ELR−	CXCL4	7.8	1100*B*						
CXC ELR+	CXCL5	8.0	0.12*D*	0.56*T*					0.66*I*
	CXCL6	7.9	0.04*R*						0.001*I*
	CXCL7	7.6	3200*B*						0.02*I*
	CXCL8	8.4	0.003*L*	0.22*T*	0.02*A*	0.038*V*	0.03*O*		0.01*I*
CXC ELR−	CXCL9	11.7	0.012*M*	0.32*T*					0.01*K*
	CXCL10	8.5	0.009*K*	0.22*T*		0.358*V*	0.008*Y*		0.06*K*
	CXCL11	8.3	0.005*S*	0.08*T*					
	CXCL12	8.0	0.3*S*	1.74*T*			0.005*Y*		
	CXCL13	10.3	0.003*U*				0.000*Y*		
	CXCL14	9.4	0.11*W*						
	CXCL16	10.1	0.26*Q*	0.02*T*					
	CXCL17	11.5		<0.005*z*					
CX3C	CX3CL1	8.5	0.1*P*			0.005*V*			
C	XCL1	10.0	0.08*P*	0.23*T*					
CC	CCL1	8.5	0.003*P*	0.04*T*					
	CCL2	8.6	0.02*E*	0.55*T*	0.01*A*	0.019*V*	0.044*Y*		
	CCL3	7.8	0.003*E*	0.19*T*			0.004*Y*		
	CCL4	7.6	0.001*P*	0.17*T*		0.001*V*			
	CCL5	7.8	1.79*E*	0.05*T*		0.009*V*	0.000*Y*		0.003*F*
	CCL7	9.0	0.003*P*	0.03*T*					
	CCL8	8.9	0.002*P*	0.05*T*					
	CCL11	8.3	0.01*P*	0.13*T*		0.007*V*			0.006*F*
	CCL13	8.6	0.026*P*	0.03*T*					
	CCL14	8.4	1.31*C*						
	CCL15	10.1	0.1*P*						
	CCL16	11.2	0.42*P*						
	CCL17	8.0	0.02*S*	0.04*T*					
	CCL18	7.8	4.23*J*						
	CCL19	8.8	0.009*P*	0.02*T*					
	CCL20	8.0	0.001*S*	0.11*T*					
	CCL21	12.2	0.01*S*	0.12*T*					
	CCL22	8.1	0.07*S*	0.07*T*					
	CCL23	11.3	0.036*P*						
	CCL24	8.8	0.025*H*						
	CCL25	14.2							
	CCL26	8.4	0.004*H*						
	CCL27	10.2	0.038*S*						
	CCL28	12.3	0.004*N*				47*G*	149*G*	24*G*

*Jones et al. (1995)*^A^*, Brandt et al. (2000)*^B^*, Nomiyama et al. (2001)*^C^*, Holven et al. (2002)*^D^*, Parissis et al. (2002)*^E^*, Bottcher et al. (2003)*^F^*, Hieshima et al. (2003)*^G^*, Kagami et al. (2003)*^H^*, Maheshwari et al. (2003)*^I^*, Struyf et al. (2003)*^J^*, Takahata et al. (2003)*^K^*, Antonelli et al. (2005)*^L^*, Chuang et al. (2005)*^M^*, Kagami et al. (2005)*^N^*, Yang et al. (2005)*^O^*, Bauer et al. (2006)*^P^*, Sheikine et al. (2006)*^Q^*, Alzoghaibi et al. (2008)*^R^*, Narbutt et al. (2009)*^S^*, Pacheco-Rodriguez et al. (2009)*^T^*, Schiffer et al. (2009)*^U^*, Carreno et al. (2010)*^V^*, Izukuri et al. (2010)*^W^*, Dong et al., 2011)*^X^*, Hernandez-Molina et al. (2011)*^Y^*, Burkhardt et al. (2012)*^z^**.

*Family and members of chemokines are listed on the left. The predicted molecular weights (MW) of mature chemokine forms are denoted in kilodaltons (kD). Reported serum or plasma concentrations of chemokines are grouped together in a single column. Italicized letter next to value designates reference source. BAL, broncholalveolar lavage. All concentrations are reported as nanomolar (nM). Blanks indicate not determined*.

During infection and inflammation, chemokine concentrations in biological fluids may be greatly increased (Luster, 1998). For example, inflamed tonsils have nanomolar concentrations of CXCL9, CXCL10, and CXCL11 whereas in normal uninflamed tonsils these chemokines are not detectable (Egesten et al., 2007). Nanomolar concentrations of CXCL9 are sufficient to inhibit *Streptococcus pyogenes* growth *in vitro*. However, in most infection models, the concentrations of chemokines detected in biological fluids are not sufficient for antimicrobial activities *in vitro*. Therefore, the combined effect of all antimicrobial chemokines present in the infection site may be needed to achieve antimicrobial effects. However, we are not aware of any data showing that antimicrobial chemokines have additive or synergistic effects. In our own unpublished studies, we had problems obtaining consistent inhibition data at low chemokine concentrations to perform combination chemokine experiments. Another possibility is that the concentration of chemokines on mucosal surfaces may be much higher than in secretions because many chemokines can bind glycosaminoglycans on cellular surfaces.

## Range of Organisms Affected by Antimicrobial Chemokines

Antimicrobial chemokines have been shown to inhibit a broad-spectrum of organisms: Gram-positive bacteria, Gram negative bacteria, and dimorphic fungi. Table 3 summarizes currently available information on this subject. In general, most antimicrobial chemokines have activity against multiple organisms. This suggests that the mechanism for antimicrobial activity is either through a conserved or non-specific pathway. All highly active antimicrobial chemokines have basic or cationic charge, but not all cationic chemokines have antimicrobial properties.

**Table 3:** **Antibacterial and antifungal properties of chemokines**.

Chemokine			MRSA		S. coag neg	*S. pyogenes*		*E. faecium*	*B. subtilis*	*L. monocytogenes*		*E. coli*		*P. aeruginosa*	*C. neoformans*	*C. glabrata*	*C. albicans*

			[Na+]		[Na+ or K+]	[Na+ or K+]		[K+]	[Na+]	[Na+]		[Na+ or K+]		[Na+ or K+]	[Na+ or K+]	[Na+]	[Na+ or K+]
Family	Member	pI	10 mM	137 mM	10 mM	10 mM	150 mM	10 mM	10 mM	10 mM	100 mM	10 mM	100 mM	10 mM	10 mM	10 mM	10 mM
CXC ELR+	CXCL1	10.09	−	−								++++*D*					
	CXCL2	10.45	+	−								+++*D*					
	CXCL3	10.15	+	−								+++*D*					
CXC ELR−	CXCL4	8.71	−	−					−*A*			−*A*			−*A*		
CXC ELR+	CXCL5	11.4	−	−		−*G*						−*B*					
	CXCL6	10.44	++	−		+*G*						+,++++*D,G*		+*G*			
	CXCL7	8.73	−	−		−*G*			+ (cleaved)*A*			+ (cleaved)*A*			+ (cleaved)*A*	−(cleaved)*A*	
	CXCL8	9.85	+	−	−*H*							−,+*D,B,H*					+*H*
CXC ELR−	CXCL9	11.1	++++	−		+*E*	+*E*			+*B*	−*B*	++++*D*	−*B*				
	CXCL10	10.77	++++	−		+*E*	+*E*			+*B*	−*B*	++++*D*	−*B*				
	CXCL11	11.1	++++	+		+*E*	+*E*			+*B*	−*B*	+++*D*	−*B*				
	CXCL12	10.48	++++	++								++++*D*					
	CXCL13	10.92	++++	+								++++*D*					
	CXCL14	10.47	−	−	+*H*							++,++++*D,H*	+++*H*				+*H*
	CXCL16	8.75	+	−													
	CXCL17	10.95										++++*j*		+*j*			+*j*
CX3C	CX3CL1	10.24	−	+								−*D,B*					
XC	XCL1	11.28	−	−								++++,−*D,B*					
CC	CCL1	10.48	++	−								++++*D*					
	CCL2	9.74	−	−								−*D,B*					
	CCL3	4.48	−	−								−*D,B*					
	CCL3L1	4.47	−	−													
	CCL4	4.47	−	+								−*B*					
	CCL5	8.61	−	−								−*D*					
	CCL7	10.33	−	+								−*D*					
	CCL8	9.88	−	−								+*D*					
	CCL11	10.74	−	−								+++*D*					
	CCL13	10.62	+++	++								+++*D*					
	CCL14	8.25	+	−													
	CCL15	8.01	−	−													
	CCL16	9.86	−	−								−*D*					
	CCL17	9.81	−	+								+++*D*					
	CCL18	9.47	+	−								++++*D*					
	CCL19	10.31	+++	−								++++*D*					
	CCL20	10.26	+++	−	+*H*	+*D*		+*D*				++++*D*	++*D*	+*D*	+*D*		+*D,H*
	CCL21	10.71	+	+								++*D*					
	CCL22	8.98	+	−								++++*D*					
	CCL23	9.09	+	+													
	CCL24	10.76	−	−													
	CCL25	10.92	+++	+								+++*D*					
	CCL26	10.85	++++	+													
	CCL27	9.07	−	−	−*H*	− in 1 mM K^+^*C*						−, +*D,H*		− in 1 mM K^+^*C*			+ in 1 mM K^+^*C*
	CCL28	10.88	+++++	−		+ in 1 mM K^+^*C*								+ in 1 mM K^+^*C*			+ in 1 mM K^+^*C*

*Krijgsveld et al. (2000)*^A^*, Cole et al. (2001)*^B^*, Hieshima et al. (2003)*^C^*, Yang et al. (2003)*^D^*, Egesten et al. (2007)*^E^*, Tohyama et al. (2007)*^F^*, Linge et al. (2008)*^G^*, Maerki et al. (2009)*^H^*, Yung et al. (2011)*^I^*, Burkhardt et al. (2012)*^j^**.

*Gram-positive organisms [methicillin-resistant *S. aureus* (MRSA), Staphylococcus coagulase negative species, *S. pyogenes*, *E. faecium*, *B. subtilis*, *L. monocytogenes*] are arranged on the left side, followed by Gram negative organisms (*E. coli*, *P. aeruginosa*) and fungi (*C. neofomans*, *C. glabrata*, *C. albicans*). Only for assays with MRSA and *E. coli* do the following symbols of inhibition apply: ++++, >80%; +++, 60–79%; ++, 40–59%; +, 20–39%; −, <20%. For all other organisms “+, ±, and −” denote >50, 20–50, and <20% antimicrobial activity, respectively. The “−, +” and “++, ++++” symbols represent discrepancies in antimicrobial properties from published literature. Italicized letter next to symbols denotes references. All MRSA data were taken from Yung et al. (2011)*^I^*. Empty boxes indicate these chemokines have not been tested. Most antimicrobial assays were performed at low salt concentration (10 mM). The CXCL7 cleaved peptide “(cleaved)” with antimicrobial properties was purified from platelet granules, and is a C-terminal processed form. The pI of each chemokine is also shown*.

Chemokines have been surveyed most comprehensively for antimicrobial activity in the case of *Staphylococcus aureus* and *Escherichia coli*. Of 45 chemokines tested, 22 were found to have some anti-staphylococcal activity (Yung et al., 2011). For *E. coli*, 25 of 33 chemokines tested had some activity (Yang et al., 2003). A report on *S. pyogenes* showed that CXCL9, CXCL10, and CXCL11 can exert antimicrobial activities even at 150 mM sodium concentration (Egesten et al., 2007). Likewise, the antimicrobial effect of CXCL9, CXCL10, and CXCL11 on *Bacillus anthracis* may also be independent of ionic concentrations (Crawford et al., 2010). These chemokines were able to inhibit both spore germination and bacillus viability in culture media. In addition to bacteria, several chemokines also have antifungal properties against *Cryptococcus neoformans* and *Candida albicans*.

## Comparison with Other Antimicrobial Proteins

Antimicrobial chemokines represent a fraction of known antimicrobial proteins.

In general, antimicrobial proteins can be divided into three different classes. The largest class consists of β-stranded proteins with four to six conserved cysteines linked by disulfide bonds. Defensins and chemokines are members of this class. Whereas chemokines typically have four conserved cysteines that form two disulfide bonds, defensins have six conserved cysteines that form three disulfide bonds to stabilize β-strands. Both β-defensins and chemokines have overall cationic charge.

The antimicrobial effect of defensins occurs at micromolar concentration and in low sodium conditions just as antimicrobial chemokines. For example, micromolar concentrations of human beta defensin two are required for activity against *E. coli* and *P. aeruginosa* (Schneider et al., 2005). Most other antimicrobial proteins also exert antimicrobial effects at micromolar concentrations. One exception is bactericidal/permeability increasing protein (BPI), which has activity against Gram negative bacteria at nanomolar concentrations (Wiesner and Vilcinskas, 2010).

The other classes of antimicrobial proteins consist of linear amphipathic helical proteins and peptides with high content of certain amino acids such as histidine, glycine, proline, or tryptophan. Since these groups have little similarity to chemokines, readers can refer to other recent reviews for details (Wiesner and Vilcinskas, 2010).

## Defining Antimicrobial and Chemotactic Domains

**Figure 3:**
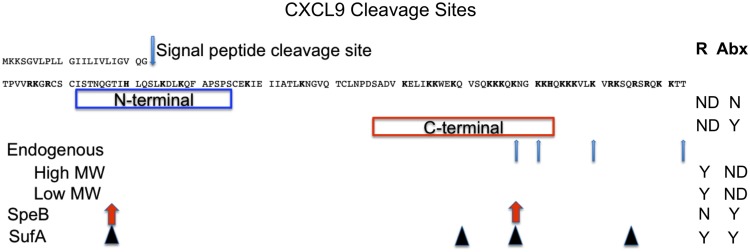
**CXCL9 cleavage sites**. CXCL9 has a signal sequence that is cleaved before secretion. Blue box indicates an N-terminal peptide corresponding to predicted beta sheet secondary structure. Red box indicates a C-terminal peptide corresponding to predicted amphipathic alpha helix secondary structure. R and Abx represent CXCR3 receptor activation and antibiotic activities, respectively. ND, N, and Y, denote not determined, negative, and positive (yes) activities, respectively. Endogenous CXCL9 has several truncated forms. These forms have been separated into two groups: high molecular weight (High MW) and low molecular weight (Low MW) groups for receptor activation. SpeB and SufA are two bacterial enzymes that cleave CXCL9. Arrows indicate where the processing sites are located.

There are no mutagenesis studies showing whether the antimicrobial and chemotactic regions of antimicrobial chemokines are linked. The best-studied antimicrobial chemokine is CXCL9. Several groups have identified CXCL9 antimicrobial activities against *S. pyogenes*, *S. aureus*, *B. anthracis*, *P. aeruginosa*, and *E. coli*. Initially discovered as the monokine induced by gamma interferon (MIG), CXCL9 is cleaved into several smaller forms endogenously (Liao et al., 1995). These smaller proteins have C-terminal truncations and are able to activate CXCR3, albeit with less potency and efficacy than full-length CXCL9. Mixtures of truncated CXCL9 proteins were separated into a high molecular weight and a low molecular weight group, presumably each group having several truncated forms. However, it is unknown whether these truncated CXCL9 forms have antimicrobial properties. A summary of functional analysis for CXCL9 is shown in Figure 3.

A 27 amino acid synthetic peptide consisting of the C-terminal region of CXCL9 has been reported to be sufficient for antimicrobial activity (red boxed). This peptide is predicted to have an amphipathic alpha helix structure. In contrast, a peptide spanning the N-terminal region of CXCL9 with beta sheet structure had no detectable antimicrobial activity (blue boxed). Both peptides have overall cationic charge. Another group tested the N-terminal (50 amino acids) and C-terminal (19 amino acids, corresponding to the alpha helix) domains of CXCL6 and found that the N-terminal fragment had higher antimicrobial properties than the C-terminal peptide (Linge et al., 2008). It may be that the whole protein is needed for full antimicrobial properties.

Streptococcal cysteine proteinase (SpeB) from *S. pyogenes* and subtilisin-like serine-proteinase (SufA) from *Finegoldia magna* can degrade full-length CXCL9 to smaller proteins (Egesten et al., 2009; Karlsson et al., 2009). By mass spectrometry, these proteins were identified to have both N-terminal and C-terminal truncations (Figure 3). Interestingly, both proteins retained antimicrobial properties for *S. pyogenes*. However there is a discrepancy between the peptides in activating CXCR3. SpeB-degraded CXCL9 cannot activate CXCR3, whereas SufA-degraded peptides can. The authors did not individually purify and test each truncated form of CXCL9. Therefore, it is possible that the least processed form of CXCL9 by SufA is responsible for receptor activation. In addition, SufA can also degrade CXCL10 and CXCL11, causing both chemokines to lose both receptor activation and antimicrobial properties. More studies will be needed to precisely determine specific regions necessary and sufficient for chemotactic and antimicrobial properties, and whether these regions overlap.

## Mechanism of Activity

By electron microscopy criteria, antimicrobial chemokines appear to induce lysis of the bacterial membrane (Hieshima et al., 2003; Egesten et al., 2007; Linge et al., 2008). Since the outer membrane of bacteria are highly anionic, charge interaction is thought to mediate initial binding (Brogden, 2005). In eukaryotic cell membranes, negatively charged phospholipids are sequestered in the inner leaflet of the lipid bilayer, and the outer leaflet is composed mostly of zwitterionic and uncharged lipids. Therefore, the lipid content of the outer membranes of eukaryotic cells are devoid of electrostatic charge (Wiesner and Vilcinskas, 2010). In contrast, both leaflets of bacterial cell membranes are enriched with acidic phospholipids such as phosphatidylglycerol and cardiolipin, making these membranes negatively charged.

The exact mechanism for antimicrobial killing is not known nor is it known whether all chemokines kill microbial targets by a common mechanism. Defensins, which have been studied in greater detail, are absorbed onto the bacterial membrane by electrostatic attraction and subsequently aggregate there to cause local membrane thinning and ion channel formation (Ganz, 2003). Rupture of bacterial membranes has been demonstrated in *S. pyogenes* for CXCL9, in *E. coli* for CXCL6, and in *P. aeruginosa* for CCL28 (Hieshima et al., 2003; Egesten et al., 2007; Linge et al., 2008).

For the bacterium responsible for anthrax, *B. anthracis*, the membrane protein FtsX has been identified as a unique target for CXCL10 (Crawford et al., 2011). The authors used a transposon-based genetic screen to identify *ftsX*, and two other genes (*BAS0651* and the cell wall autolysin *lytE*) to be critical for resistance against CXCL10. *B. anthracis* genetically deficient in *ftsX* could not bind CXCL10 as assessed by transmission electron microscopy. FtsX is a cell division ABC transporter that is conserved among Gram-positive and Gram negative bacteria. Comparative protein analysis of FtsX with CXCR3, the receptor for CXCL10, showed a 27-residue region of FtsX that has 45% similarity with the N-terminal chemokine-binding domain of CXCR3. This is low, and how FtsX actually functions in antimicrobial chemokine resistance remains to be determined.

If charge–charge interaction were the sole means of chemokine-binding and antimicrobial action, chemokines would not be predicted to have antifungal properties since fungi have eukaryotic membranes. Histatin, a known antifungal peptide, inhibits *C. albicans* by binding to fungal mitochondria and inhibiting respiration by inducing reactive oxygen species (ROS) formation (Helmerhorst et al., 2001). The level of induced ROS correlated with fungicidal activity. However, it is not known whether antimicrobial chemokines act through similar pathways. The chemokine CCL28 has been shown to cause membrane disruption in *C. albicans* by scanning electron microscopy (Hieshima et al., 2003). How it binds to the fungal membrane and the domain responsible for antifungal activities has not been determined.

By whatever mechanisms chemokines kill pathogens, they do so rapidly. CXCL6 can disrupt liposome membranes within minutes, and pathogens treated with CCL28 show numerous surface blebs within 1 h (Hieshima et al., 2003; Linge et al., 2008). It is unclear whether gene activation or signal transduction needs to occur for antimicrobial chemokines to kill targets. Unlike traditional antibiotics, which work best on dividing organisms, antimicrobial proteins typically can exert effects on non-dividing organisms (Wiesner and Vilcinskas, 2010).

The salt dependence seen with antimicrobial chemokines is typical of most antimicrobial proteins. With the exception of human beta defensin 3, all human defensins exert antimicrobial properties at ∼10 mM Na+. Killing of pathogens is thought to occur *in vivo* even in host environments with >10 mM Na+ because defensins are packed in high concentration within granules. Activation and degranulation of leukocytes by pathogens would produce very high local concentrations of defensins for optimal antimicrobial effects. In addition, two recent reports have shown that the antimicrobial activity of chemokines is not dependent on sodium concentration for certain bacteria. In particular, CXCL9, CXCL10, and CXCL11 are able to inhibit growth of *S. pyogenes* even in 150 mM sodium chloride solutions. The same chemokines could also inhibit *B. anthracis* germination and growth in culture media with serum.

## Inhibition of Antimicrobial Chemokines by Bacteria

In addition to degrading chemokines with SpeB, *S. pyogenes* produces additional factors that inhibit antimicrobial chemokines. Streptococcal interleukin-8 inactivating cell envelope protease (SpyCEP) cleaves CXCL1, 2, 6, and 8 (Zinkernagel et al., 2008), and Streptococcal inhibitor of complement (SIC) binds CXCL9 to inhibit its antimicrobial properties (Egesten et al., 2007). As mentioned previously, the bacterium *F. magna* expresses a subtilisin-like serine-proteinase (SufA) that partially degrades CXCL9, abolishing its antimicrobial activity (Karlsson et al., 2009). Therefore, several bacteria have developed means to degrade antimicrobial chemokines and evade host defense.

## *In vivo* Evidence for Antimicrobial Properties of Chemokines

Many inflammatory chemokines with antimicrobial properties are induced during inflammatory or infectious processes. It is unclear to what extent control of pathogens depends on direct antimicrobial activities of chemokines versus recruitment of leukocytes. One study demonstrated that neutralization of either CXCL9 or CXCL10 but not CXCR3 (the receptor for CXCL9 and CXCL10) increased susceptibility of C57BL/6 mice to *B. anthracis* (Crawford et al., 2011). Increased mortality was shown to arise from extrapulmonary dissemination of bacteria to kidneys, spleen, and liver and from toxemia with lethal factor. However, the bacterial load at the site of inoculation, the lung, was similar between treated and untreated mice.

We are not aware of genetic mutations in chemokine genes leading to increased susceptibility to infectious diseases in humans. However, there is persuasive evidence that the chemokine system mediates control of pathogens in mammals, including humans. For example, a form of congenital neutropenia, WHIM syndrome, arises from gain-of-function mutations truncating the C-tail of CXCR4 (Hernandez et al., 2003). Patients with WHIM syndrome have recurrent bacterial infections, especially in the sino-pulmonary tract, apparently caused by neutropenia and hypogammaglobulinemia (Kawai and Malech, 2009). However, this susceptibility appears to be due to leukocyte trafficking defects, not direct antimicrobial effects of chemokines.

## Future Directions

There is no doubt that many chemokines have antimicrobial properties *in vitro*. However, the *in vivo* significance of chemokines as antimicrobial agents is still unclear. Direct antimicrobial activity will depend on the chemokine concentration and the environmental context between host and pathogen. In theory, chemokines in mucosal surfaces might inhibit colonization of certain bacteria in the host since antibiotic usage has clearly been shown to alter normal flora. New studies will be needed to determine whether lack of constitutive chemokines affect skin and mucosal flora.

Studying the domains important for antimicrobial activities might lead to more potent antimicrobial peptides. Cationic charge and amphipathic alpha helix secondary structure appears to be important for antimicrobial properties. However, with the great diversity of antimicrobial peptides there has been neither consensus primary sequence nor a predominant tertiary structure. This makes molecular modeling to generate more potent synthetic antimicrobial proteins difficult. There is also the issue of unwanted side effects. Several potent antimicrobial peptides have systemic toxicity because they lyse red blood cells as well as pathogen membranes (Wiesner and Vilcinskas, 2010). In addition, we have found that many chemokines can induce the release of a virulence factor, protein A, in *S. aureus* (Yung et al., 2011). This potentially would harm the host if bacteria deploy virulence factors in response to chemokines. There is also evidence that subinhibitory concentrations of aminoglycoside antibiotics induce biofilm formation (Hoffman et al., 2005). Antibiotics trigger a protective response by bacteria to form biofilms. Bacteria in biofilms are very difficult to eradicate and are generally thousands of times more resistant to antimicrobial therapy than planktonic bacteria. In the same manner, antimicrobial proteins might trigger a protective response by bacteria to form biofilms.

Currently, there are no antimicrobial proteins in clinical use. The antimicrobial peptide MSI-78 or pexiganan completed a phase III clinical trial for the treatment of diabetic foot ulcers. MSI-78 was originally derived from magainan, an antimicrobial peptide found on the skin of frogs (Zasloff, 1987). However, it failed to receive approval from the FDA on the grounds that efficacy was not sufficiently demonstrated. Few companies are actively developing antimicrobial proteins as therapeutics. Both MBI-226 (a 12 amino acid peptide developed for the prevention of catheter-related bloodstream infections) and Neuprex (a recombinant fragment of bactericidal/permeability increasing protein developed for meningococcal meningitis) finished phase III clinical trials and never made it to market. It is unclear how antimicrobial proteins will be used in the clinics. Topical formulations to treat skin infections may be most applicable but effective topical antiseptics such as chlorhexidine already exist. To treat invasive infections, an injectable or intravenous form would be needed since proteins would most likely be degraded in the gastrointestinal tract. If developed, such potential broad-spectrum antimicrobial peptides might be useful in the setting of life-threatening infections caused by multi-drug resistant organisms.

Even though antimicrobial chemokines have been shown to generally disrupt bacterial membranes, unique targets on pathogens may actually mediate killing. As mentioned previously, the cell membrane protein FtsX in *B. anthracis* binds CXCL10 specifically and mediates antimicrobial chemokine resistance. Understanding this pathway and determining whether the same or related pathways exist in other organisms may lead to new antibiotics.

Genome-wide association studies have not linked increased infection susceptibility to single nucleotide polymorphism in chemokine genes. This could imply either no association, a weak association that has not been analyzed, or redundancy of the antimicrobial protein system. Further genetic studies in mice will be able to address whether genetic deficiencies in chemokines correlate with pathogen control, especially when compared with their respective chemokine receptor deficient controls.

## Conclusion

In conclusion, the utility of chemokines likely extends beyond leukocyte recruitment. Understanding domains essential for antimicrobial activities in antimicrobial chemokines could lead to more potent antimicrobial peptides. In addition, specific pathways may be involved in pathogen recognition of antimicrobial chemokines. Much work is needed to determine whether antimicrobial chemokines affect susceptibility to infection in humans, how chemokines kill microbes, and whether antimicrobial chemokines can be use to treat infections.

## Conflict of Interest Statement

The authors declare that the research was conducted in the absence of any commercial or financial relationships that could be construed as a potential conflict of interest.

## References

[B1] Alzoghaibi M. A. Al-Mofleh I. A. Al-Jebreen A. M. (2008). Neutrophil chemokines GCP-2 and GRO-alpha in patients with inflammatory bowel disease. J. Dig. Dis. 9, 144–148 10.1111/j.1751-2980.2008.00336.x 18956592

[B2] Antonelli A. Rotondi M. Fallahi P. Romagnani P. Ferrari S. M. Ferrannini E. Serio M. (2005). Age-dependent changes in CXC chemokine ligand 10 serum levels in euthyroid subjects. J. Interferon Cytokine Res. 25, 547–552 10.1089/jir.2005.25.547 16181055

[B3] Bauer J. W. Baechler E. C. Petri M. Batliwalla F. M. Crawford D. Ortmann W. A. Espe K. J. Li W. Patel D. D. Gregersen P. K. Behrens T. W. (2006). Elevated serum levels of interferon-regulated chemokines are biomarkers for active human systemic lupus erythematosus. PLoS Med. 3, e491 10.1371/journal.pmed.0030491 17177599 PMC1702557

[B4] Berkhout T. A. Sarau H. M. Moores K. White J. R. Elshourbagy N. Appelbaum E. Reape R. J. Brawner M. Makwana J. Foley J. J. Schmidt D. B. Imburgia C. McNulty D. Matthews J. O’Donnell K. O’Shannessy D. Scott M. Groot P. H. MacPhee C. (1997). Cloning, in vitro expression, and functional characterization of a novel human CC chemokine of the monocyte chemotactic protein (MCP) family (MCP-4) that binds and signals through the CC chemokine receptor 2B. J. Biol. Chem. 272, 16404–16413 10.1074/jbc.272.26.16404 9195948

[B5] Bottcher M. F. Jenmalm M. C. Bjorksten B. (2003). Cytokine, chemokine and secretory IgA levels in human milk in relation to atopic disease and IgA production in infants. Pediatr. Allergy Immunol. 14, 35–41 10.1034/j.1399-3038.2003.02119.x 12603709

[B6] Brandt E. Petersen F. Ludwig A. Ehlert J. E. Bock L. Flad H. D. (2000). The beta-thromboglobulins and platelet factor 4: blood platelet-derived CXC chemokines with divergent roles in early neutrophil regulation. J. Leukoc. Biol. 67, 471–478 10770278 10.1002/jlb.67.4.471

[B7] Brogden K. A. (2005). Antimicrobial peptides: pore formers or metabolic inhibitors in bacteria? Nat. Rev. Microbiol. 3, 238–250 10.1038/nrmicro1098 15703760

[B8] Bulmer M. G. Forwell G. D. (1956). The concentration of sodium in thermal sweat. J. Physiol. (Lond.) 132, 115–122 13320376 10.1113/jphysiol.1956.sp005506 PMC1363543

[B9] Burkhardt A. M. Tai K. P. Flores-Guiterrez J. P. Vilches-Cisneros N. Kamdar K. Barbosa-Quintana O. Valle-Rios R. Hevezi P. A. Zuniga J. Selman M. Ouellette A. J. Zlotnik A. (2012). CXCL17 is a mucosal chemokine elevated in idiopathic pulmonary fibrosis that exhibits broad antimicrobial activity. J. Immunol. 188, 6399–6406 10.4049/jimmunol.1102903 22611239 PMC3370106

[B10] Carreno E. Enriquez-De-Salamanca A. Teson M. Garcia-Vazquez C. Stern M. E. Whitcup S. M. Calonge M. (2010). Cytokine and chemokine levels in tears from healthy subjects. Acta Ophthalmol. 88, e250–e258 10.1111/j.1755-3768.2010.01978.x 20738261

[B11] Cavaliere F. Masieri S. Proietti R. Magalini S. I. (1988). pH and electrolytes in nasal secretum of intensive care unit patients. Resuscitation 16, 133–137 10.1016/0300-9572(88)90078-0 2839883

[B12] Cavaliere F. Masieri S. Vagnoni S. Proietti R. Magalini S. I. (1989). Airway secretion electrolytes: reflection of water and salt states of the body. Crit. Care Med. 17, 891–894 10.1097/00003246-198909000-00010 2766762

[B13] Chicharro J. L. Legido J. C. Alvarez J. Serratosa L. Bandres F. Gamella C. (1994). Saliva electrolytes as a useful tool for anaerobic threshold determination. Eur. J. Appl. Physiol. Occup. Physiol. 68, 214–218 10.1007/BF00376769 8039517

[B14] Chuang Y. H. Lian Z. X. Cheng C. M. Lan R. Y. Yang G. X. Moritoki Y. Chiang B. L. Ansari A. A. Tsuneyama K. Coppel R. L. Gershwin M. E. (2005). Increased levels of chemokine receptor CXCR3 and chemokines IP-10 and MIG in patients with primary biliary cirrhosis and their first degree relatives. J. Autoimmun. 25, 126–132 10.1016/j.jaut.2005.08.009 16243485

[B15] Cole A. M. Ganz T. Liese A. M. Burdick M. D. Liu L. Strieter R. M. (2001). Cutting edge: IFN-inducible ELR-CXC chemokines display defensin-like antimicrobial activity. J. Immunol. 167, 623–627 11441062 10.4049/jimmunol.167.2.623

[B16] Crawford M. A. Burdick M. D. Glomski I. J. Boyer A. E. Barr J. R. Mehrad B. Strieter R. M. Hughes M. A. (2010). Interferon-inducible cxc chemokines directly contribute to host defense against inhalational anthrax in a murine model of infection. PLoS Pathog. 6, e1001199 10.1371/journal.ppat.1001199 21124994 PMC2987825

[B17] Crawford M. A. Lowe D. E. Fisher D. J. Stibitz S. Plaut R. D. Beaber J. W. Zemansky J. Mehrad B. Glomski I. J. Strieter R. M. Hughes M. A. (2011). Identification of the bacterial protein FtsX as a unique target of chemokine-mediated antimicrobial activity against *Bacillus anthracis*. Proc. Natl. Acad. Sci. U.S.A. 108, 17159–17164 10.1073/pnas.1108495108 21949405 PMC3193227

[B18] Dong Q. M. Zhang J. Q. Li Q. Bracher J. C. Hendricks D. T. Zhao X. H. (2011). Clinical significance of serum expression of GRObeta in esophageal squamous cell carcinoma. World J. Gastroenterol. 17, 2658–2662 10.3748/wjg.v17.i9.1204 21677836 PMC3110930

[B19] Egesten A. Eliasson M. Johansson H. M. Olin A. I. Morgelin M. Mueller A. Pease J. E. Frick I. M. Bjorck L. (2007). The CXC chemokine MIG/CXCL9 is important in innate immunity against *Streptococcus pyogenes*. J. Infect. Dis. 195, 684–693 10.1086/510857 17262710

[B20] Egesten A. Olin A. I. Linge H. M. Yadav M. Morgelin M. Karlsson A. Collin M. (2009). SpeB of *Streptococcus pyogenes* differentially modulates antibacterial and receptor activating properties of human chemokines. PLoS ONE 4, e4769 10.1371/journal.pone.0004769 19274094 PMC2652026

[B21] Ganz T. (2003). Defensins: antimicrobial peptides of innate immunity. Nat. Rev. Immunol. 3, 710–720 10.1038/nri1180 12949495

[B22] Helmerhorst E. J. Troxler R. F. Oppenheim F. G. (2001). The human salivary peptide histatin 5 exerts its antifungal activity through the formation of reactive oxygen species. Proc. Natl. Acad. Sci. U.S.A. 98, 14637–14642 10.1073/pnas.141366998 11717389 PMC64734

[B23] Hernandez P. A. Gorlin R. J. Lukens J. N. Taniuchi S. Bohinjec J. Francois F. Klotman M. E. Diaz G. A. (2003). Mutations in the chemokine receptor gene CXCR4 are associated with WHIM syndrome, a combined immunodeficiency disease. Nat. Genet. 34, 70–74 10.1038/ng1149 12692554

[B24] Hernandez-Molina G. Michel-Peregrina M. Hernandez-Ramirez D. F. Sanchez-Guerrero J. Llorente L. (2011). Chemokine saliva levels in patients with primary Sjogren’s syndrome, associated Sjogren’s syndrome, pre-clinical Sjogren’s syndrome and systemic autoimmune diseases. Rheumatology (Oxford) 50, 1288–1292 10.1093/rheumatology/ker196 21330342

[B25] Hieshima K. Ohtani H. Shibano M. Izawa D. Nakayama T. Kawasaki Y. Shiba F. Shiota M. Katou F. Saito T. Yoshie O. (2003). CCL28 has dual roles in mucosal immunity as a chemokine with broad-spectrum antimicrobial activity. J. Immunol. 170, 1452–1461 12538707 10.4049/jimmunol.170.3.1452

[B26] Hoffman L. R. D’Argenio D. A. Maccoss M. J. Zhang Z. Jones R. A. Miller S. I. (2005). Aminoglycoside antibiotics induce bacterial biofilm formation. Nature 436, 1171–1175 10.1038/436189a 16121184

[B27] Holven K. B. Aukrust P. Holm T. Ose L. Nenseter M. S. (2002). Folic acid treatment reduces chemokine release from peripheral blood mononuclear cells in hyperhomocysteinemic subjects. Arterioscler. Thromb. Vasc. Biol. 22, 699–703 10.1161/01.ATV.0000013288.35930.90 11950713

[B28] Izukuri K. Ito S. Nozaki N. Yajima N. Iwamiya M. Kawahara S. Suzuki K. Kubota E. Hata R. (2010). Determination of serum BRAK/CXCL14 levels in healthy volunteers. Lab. med. 41, 478–482 10.1309/LMQOXCQEF7ZXIIUK

[B29] Jones A. P. Webb L. M. Anderson A. O. Leonard E. J. Rot A. (1995). Normal human sweat contains interleukin-8. J. Leukoc. Biol. 57, 434–437 7884315 10.1002/jlb.57.3.434

[B30] Kagami S. Kakinuma T. Saeki H. Tsunemi Y. Fujita H. Nakamura K. Takekoshi T. Kishimoto M. Mitsui H. Torii H. Komine M. Asahina A. Tamaki K. (2003). Significant elevation of serum levels of eotaxin-3/CCL26, but not of eotaxin-2/CCL24, in patients with atopic dermatitis: serum eotaxin-3/CCL26 levels reflect the disease activity of atopic dermatitis. Clin. Exp. Immunol. 134, 309–313 10.1046/j.1365-2249.2003.02273.x 14616792 PMC1808865

[B31] Kagami S. Kakinuma T. Saeki H. Tsunemi Y. Fujita H. Sasaki K. Nakamura K. Takekoshi T. Kishimoto M. Mitsui H. Komine M. Asahina A. Tamaki K. (2005). Increased serum CCL28 levels in patients with atopic dermatitis, psoriasis vulgaris and bullous pemphigoid. J. Invest. Dermatol. 124, 1088–1090 10.1111/j.0022-202X.2005.23700.x 15854059

[B32] Kaiser D. Songo-Williams R. Drack E. (1974). Hydrogen ion and electrolyte excretion of the single human sweat gland. Pflugers Arch. 349, 63–72 10.1007/BF00587917 4857832

[B33] Karlsson C. Eliasson M. Olin A. I. Morgelin M. Karlsson A. Malmsten M. Egesten A. Frick I. M. (2009). SufA of the opportunistic pathogen finegoldia magna modulates actions of the antibacterial chemokine MIG/CXCL9, promoting bacterial survival during epithelial inflammation. J. Biol. Chem. 284, 29499–29508 10.1074/jbc.M109.025957 19628464 PMC2785583

[B34] Kawai T. Malech H. L. (2009). WHIM syndrome: congenital immune deficiency disease. Curr. Opin. Hematol. 16, 20–26 10.1097/MOH.0b013e32831ac557 19057201 PMC2673024

[B35] Koo W. W. Gupta J. M. (1982). Breast milk sodium. Arch. Dis. Child. 57, 500–502 10.1136/adc.57.6.447 7103538 PMC1627692

[B36] Krijgsveld J. Zaat S. A. Meeldijk J. Van Veelen P. A. Fang G. Poolman B. Brandt E. Ehlert J. E. Kuijpers A. J. Engbers G. H. Feijen J. Dankert J. (2000). Thrombocidins, microbicidal proteins from human blood platelets, are C-terminal deletion products of CXC chemokines. J. Biol. Chem. 275, 20374–20381 10.1074/jbc.275.27.20374 10877842

[B37] Lew H. Yun Y. S. Lee S. Y. (2005). Electrolytes and electrophoretic studies of tear proteins in tears of patients with nasolacrimal duct obstruction. Ophthalmologica 219, 142–146 10.1159/000085246 15947499

[B38] Liao F. Rabin R. L. Yannelli J. R. Koniaris L. G. Vanguri P. Farber J. M. (1995). Human Mig chemokine: biochemical and functional characterization. J. Exp. Med. 182, 1301–1314 10.1084/jem.182.5.1301 7595201 PMC2192190

[B39] Linge H. M. Collin M. Nordenfelt P. Morgelin M. Malmsten M. Egesten A. (2008). The human CXC chemokine granulocyte chemotactic protein 2 (GCP-2)/CXCL6 possesses membrane-disrupting properties and is antibacterial. Antimicrob. Agents Chemother. 52, 2599–2607 10.1128/AAC.00028-08 18443119 PMC2443903

[B40] Luster A. D. (1998). Chemokines – chemotactic cytokines that mediate inflammation. N. Engl. J. Med. 338, 436–445 10.1056/NEJM199802123380706 9459648

[B41] Maerki C. Meuter S. Liebi M. Muhlemann K. Frederick M. J. Yawalkar N. Moser B. Wolf M. (2009). Potent and broad-spectrum antimicrobial activity of CXCL14 suggests an immediate role in skin infections. J. Immunol. 182, 507–514 19109182 10.4049/jimmunol.182.1.507

[B42] Maheshwari A. Christensen R. D. Calhoun D. A. (2003). ELR+ CXC chemokines in human milk. Cytokine 24, 91–102 10.1016/j.cyto.2003.07.002 14581003

[B43] Moritz M. L. (2008). Urine sodium composition in ambulatory healthy children: hypotonic or isotonic? Pediatr. Nephrol. 23, 955–957 10.1007/s00467-008-0757-6 18288498

[B44] Murphy P. M. (2008). Chemokines and Chemokine Receptors. Philadelphia, PA: Elsevier

[B45] Murphy P. M. Baggiolini M. Charo I. F. Hebert C. A. Horuk R. Matsushima K. Miller L. H. Oppenheim J. J. Power C. A. (2000). International union of pharmacology. XXII. Nomenclature for chemokine receptors. Pharmacol. Rev. 52, 145–176 10699158

[B46] Narbutt J. Lesiak A. Sysa-Jedrzeiowska A. Zakrzewski M. Bogaczewicz J. Stelmach I. Kuna P. (2009). The imbalance in serum concentration of Th-1- and Th-2-derived chemokines as one of the factors involved in pathogenesis of atopic dermatitis. Mediators Inflamm. 2009, 269541 10.1155/2009/269541 19639049 PMC2715822

[B47] Nomiyama H. Hieshima K. Nakayama T. Sakaguchi T. Fujisawa R. Tanase S. Nishiura H. Matsuno K. Takamori H. Tabira Y. Yamamoto T. Miura R. Yoshie O. (2001). Human CC chemokine liver-expressed chemokine/CCL16 is a functional ligand for CCR1, CCR2 and CCR5, and constitutively expressed by hepatocytes. Int. Immunol. 13, 1021–1029 10.1093/intimm/13.8.1021 11470772

[B48] Olson T. S. Ley K. (2002). Chemokines and chemokine receptors in leukocyte trafficking. Am. J. Physiol. Regul. Integr. Comp. Physiol. 283, R7–R28 12069927 10.1152/ajpregu.00738.2001

[B49] Pacheco-Rodriguez G. Kumaki F. Steagall W. K. Zhang Y. Ikeda Y. Lin J. P. Billings E. M. Moss J. (2009). Chemokine-enhanced chemotaxis of lymphangioleiomyomatosis cells with mutations in the tumor suppressor TSC2 gene. J. Immunol. 182, 1270–1277 19155472 10.4049/jimmunol.182.3.1270 PMC2947111

[B50] Parissis J. T. Korovesis S. Giazitzoglou E. Kalivas P. Katritsis D. (2002). Plasma profiles of peripheral monocyte-related inflammatory markers in patients with arterial hypertension. Correlations with plasma endothelin-1. Int. J. Cardiol. 83, 13–21 10.1016/S0167-5273(02)00021-9 11959378

[B51] Schiffer L. Kumpers P. Davalos-Misslitz A. M. Haubitz M. Haller H. Anders H. J. Witte T. Schiffer M. (2009). B-cell-attracting chemokine CXCL13 as a marker of disease activity and renal involvement in systemic lupus erythematosus (SLE). Nephrol. Dial. Transplant. 24, 3708–3712 10.1093/ndt/gfp343 19602475

[B52] Schneider J. J. Unholzer A. Schaller M. Schafer-Korting M. Korting H. C. Routsias J. G. Karagounis P. Parvulesku G. Legakis N. J. Tsakris A. (2005). Human defensins in vitro bactericidal activity of human beta-defensin 2 against nosocomial strains. J. Mol. Med. (Berl.). 83, 587–595 10.1007/s00109-005-0657-1 15821901

[B53] Sheikine Y. Bang C. S. Nilsson L. Samnegard A. Hamsten A. Jonasson L. Eriksson P. Sirsjo A. (2006). Decreased plasma CXCL16/SR-PSOX concentration is associated with coronary artery disease. Atherosclerosis 188, 462–466 10.1016/j.atherosclerosis.2005.11.025 16378611

[B54] Shiau Y. F. (1987). Clinical and laboratory approaches to evaluate diarrheal disorders. Crit. Rev. Clin. Lab. Sci. 25, 43–69 10.3109/10408368709105877 3301211

[B55] Singh S. P. Zhang H. H. Foley J. F. Hedrick M. N. Farber J. M. (2008). Human T cells that are able to produce IL-17 express the chemokine receptor CCR6. J. Immunol. 180, 214–221 18097022 10.4049/jimmunol.180.1.214

[B56] Siveke J. T. Hamann A. (1998). T helper 1 and T helper 2 cells respond differentially to chemokines. J. Immunol. 160, 550–554 9551886

[B57] Struyf S. Schutyser E. Gouwy M. Gijsbers K. Proost P. Benoit Y. Opdenakker G. Van Damme J. Laureys G. (2003). PARC/CCL18 is a plasma CC chemokine with increased levels in childhood acute lymphoblastic leukemia. Am. J. Pathol. 163, 2065–2075 10.1016/S0002-9440(10)63564-X 14578205 PMC1892433

[B58] Takahata Y. Takada H. Nomura A. Nakayama H. Ohshima K. Hara T. (2003). Detection of interferon-gamma-inducible chemokines in human milk. Acta Paediatr. 92, 659–665 10.1111/j.1651-2227.2003.tb00595.x 12856973

[B59] Tohyama M. Sayama K. Komatsuzawa H. Hanakawa Y. Shirakata Y. Dai X. Yang L. Tokumaru S. Nagai H. Hirakawa S. Sugai M. Hashimoto K. (2007). CXCL16 is a novel mediator of the innate immunity of epidermal keratinocytes. Int. Immunol. 19, 1095–1102 10.1093/intimm/dxm083 17855433

[B60] Wack R. P. Lien E. L. Taft D. Roscelli J. D. (1997). Electrolyte composition of human breast milk beyond the early postpartum period. Nutrition 13, 774–777 10.1016/S0899-9007(97)00187-1 9290089

[B61] Wheeler H. O. Ramos O. L. Whitlock R. T. (1960). Electrolyte excretion in bile. Circulation 21, 988–996 10.1161/01.CIR.21.5.988 13844287

[B62] Wiesner J. Vilcinskas A. (2010). Antimicrobial peptides: the ancient arm of the human immune system. Virulence 1, 440–464 10.4161/viru.1.5.12983 21178486

[B63] Yang C. Y. Brooks E. Li Y. Denny P. Ho C. M. Qi F. Shi W. Wolinsky L. Wu B. Wong D. T. Montemagno C. D. (2005). Detection of picomolar levels of interleukin-8 in human saliva by SPR. Lab. Chip 5, 1017–1023 10.1039/b502121a 16175255

[B64] Yang D. Chen Q. Hoover D. M. Staley P. Tucker K. D. Lubkowski J. Oppenheim J. J. (2003). Many chemokines including CCL20/MIP-3alpha display antimicrobial activity. J. Leukoc. Biol. 74, 448–455 10.1189/jlb.0103024 12949249

[B65] Yeaman M. R. (1997). The role of platelets in antimicrobial host defense. Clin. Infect. Dis. 25, 951–968 [quiz 969–970]. 10.1086/516120 9402338

[B66] Yung S. C. Parenti D. Murphy P. M. (2011). Host chemokines bind to *Staphylococcus aureus* and stimulate protein A release. J. Biol. Chem. 286, 5069–5077 10.1074/jbc.M110.195180 21138841 PMC3037618

[B67] Zasloff M. (1987). Magainins, a class of antimicrobial peptides from Xenopus skin: isolation, characterization of two active forms, and partial cDNA sequence of a precursor. Proc. Natl. Acad. Sci. U.S.A. 84, 5449–5453 10.1073/pnas.84.15.5449 3299384 PMC298875

[B68] Zinkernagel A. S. Timmer A. M. Pence M. A. Locke J. B. Buchanan J. T. Turner C. E. Mishalian I. Sriskandan S. Hanski E. Nizet V. (2008). The IL-8 protease SpyCEP/ScpC of group A Streptococcus promotes resistance to neutrophil killing. Cell Host Microbe 4, 170–178 10.1016/j.chom.2008.07.002 18692776 PMC2631432

